# Task Attention Facilitates Learning of Task-Irrelevant Stimuli

**DOI:** 10.1371/journal.pone.0035946

**Published:** 2012-04-26

**Authors:** Tsung-Ren Huang, Takeo Watanabe

**Affiliations:** Department of Psychology, Boston University, Boston, Massachusetts, United States of America; Northwestern University, United States of America

## Abstract

Attention plays a fundamental role in visual learning and memory. One highly established principle of visual attention is that the harder a central task is, the more attentional resources are used to perform the task and the smaller amount of attention is allocated to peripheral processing because of limited attention capacity. Here we show that this principle holds true in a dual-task setting but not in a paradigm of task-irrelevant perceptual learning. In Experiment 1, eight participants were asked to identify either bright or dim number targets at the screen center and to remember concurrently presented scene backgrounds. Their recognition performances for scenes paired with dim/hard targets were worse than those for scenes paired with bright/easy targets. In Experiment 2, eight participants were asked to identify either bright or dim letter targets at the screen center while a task-irrelevant coherent motion was concurrently presented in the background. After five days of training on letter identification, participants improved their motion sensitivity to the direction paired with hard/dim targets improved but not to the direction paired with easy/bright targets. Taken together, these results suggest that task-irrelevant stimuli are not subject to the attentional control mechanisms that task-relevant stimuli abide.

## Introduction

Attention is a major area of investigation in psychology and neuroscience. It can be defined as a cognitive process that allocates limited-capacity brain resources selectively to one aspect of sensory information while ignoring others [1]. A classic example of attention is our ability of focusing on a particular conversation in a party where a number of people are talking simultaneously [2].

In the visual domain, a spotlight or zoom-lens metaphor is prevalently used to characterize selective attention. According to the spotlight model [3], attention can be directed to various spatial locations across a visual scene like a spotlight with a focus, a fringe, and a margin. Information within the focused area is processed more efficiently. The zoom-lens model further proposes the attentional spotlight as a variable aperture like zoom lens [4], and suggests a trade-off between the spotlight size and processing efficiency [5].

Specifically, our visual system can process task-relevant information more efficiently when attention zooms in to a smaller area. On the contrary, when attention zooms out, visual analysis over a larger region tends to be coarse [6]–[7]. Consistent with these theories, research of functional visual field found shrinkage of the functional visual field size when foveal recognition difficulty increased [8]. It has also been reported that difficult task-relevant components diminish task-irrelevant processing [9]–[11]. For example, increasing the perceptual difficulty of a foveal face task attenuated processing of task-irrelevant background scenes [11].

In the current study, we examined whether such a widely accepted principle of attention could be a general account for visual learning and memory. If the principle of attention always holds true, every difficult central task should limit processing and learning of stimuli in the visual periphery.

## Materials and Methods

### Participants

Eight college students at Boston University participated in Experiment 1 for class credits. Another group of eight participants were recruited for money payments in Experiment 2. All participants who ranged in age from 18 to 24 years had normal or corrected-to-normal vision and were naïve regarding the purpose of the experiment.

### Stimuli and procedure

The stimuli were presented using Psychophysics Toolbox [12] for MATLAB® (The MathWorks, Natick, MA) on a Macintosh computers. All stimuli were viewed binocularly at a distance of 57 cm on a LCD monitor (34-cm wide in Experiment 1 and 37.5-cm wide in Experiment 2) that was set to a resolution of 1024×768 and a refresh rate of 60 Hz. A chin-rest was used to stabilize the head. The participants used a computer keyboard to make responses.

### Experiment 1

The first experiment used a dual-task design to test how multiple task-relevant stimuli compete for attention and storage into visual memory.

The backgrounds of all displays were gray (50.2 cd/m^2^). Display items consisted of 1000 700×700 pixel (21.1 degrees of visual angle) images of animals, scenes, objects, textures, and abstract patterns. These high-resolution images were included in the CorelDRAW® Graphics Suite X5, and down-sampled to 700×700 pixels of resolution.

**Figure 1:**
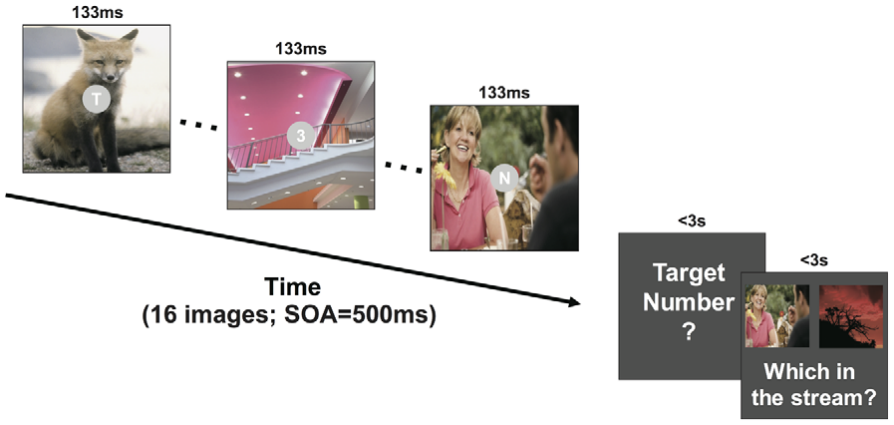
In a RSVP trial, an observer identified a flashed number while being exposed to background scenes. Target numbers and distractor letters at the screen center were equally bright or dim within a block. Their luminance was alternated across blocks. SOA = Stimulus Onset Asynchrony.

Display items during the experiment were sampled with replacement from all the 1000 scenes for rapid serial visual presentation (RSVP). In each RSVP trial, participants were shown 16 of these scenes at 133 ms per scene, followed by a blank ISI of 367 ms for a SOA of 500 ms (Figure 1). In addition, 15 different English letters and 1 Arabic number were randomly chosen, each shown on a 1° gray disk (50.2 cd/m^2^) at the center of a scene.

Each observer participated in only one session, which comprised a 5-minute practice followed by a 45-minute experiment in a dimly lit room. A practice trial was identical to that in the real experiment except that high- or low-pitch tones were provided at the end of each trial to indicate correct or incorrect answers, respectively. Between trials, a blank gray screen was presented for 1 s.

### Number Identification Task

The number identification task required participants to identify an Arabic number that was designated as the target in a letter RSVP stream. A target could only appear at serial positions 5–12 in the 16-item RSVP. At the end of each trial, participants were asked to type the number within 3 seconds.

To avoid confusable cases, distractor letters were chosen from the English alphabet except (‘B’, ‘I’, ‘T’, ‘Z’), and target numbers were chosen from 1 to 9. Therefore, the letter identification was essentially a 9-alternative forced-choice task.

Two conditions of task difficulty were tested in a within-subject design. The luminance of number/letters was high (105.4 cd/m^2^) in the easy condition, and low in the hard condition (53.9 cd/m^2^). For each participant, 200 trials were grouped into 5 blocks for each condition, and two blocked conditions were interleaved with a self-paced break in between adjacent blocks.

### Memory Recognition Task

After the response for letter identification, participants were further asked to indicate, within 3 seconds, which of the two prompted scenes was just presented as a background in the RSVP stream. The inside-RSVP probe had an equal chance of being a distractor background or a target background, and the outside-RSVP probe was randomly sampled from the remaining 984 images.

### Experiment 2

The second experiment further inspected attentional control during visual perceptual learning, which is defined as long-term performance enhancement as a result of visual experiences [13], [14]. In particular, we used the paradigm of task-irrelevant perceptual learning (TIPL) by which perceptual learning could result from passive exposure to a subliminal task-irrelevant feature presented in the visual periphery while a central task was performed [15]–[17].

We adapted the standard TIPL training and testing procedure [15] into a simpler design where only two background motion directions were studied for two experimental conditions concerning task difficulty. In addition, unlike a RSVP design in Experiment 1 and other TIPL experiments, no distractors were presented within a trial in Experiment 2 for increasing the total exposure time of target backgrounds.

For all tasks, motion was created by 3 interleaved sets of random dots with the Movshon/Newsome algorithm [18]. White dots (108.4 cd/m^2^) moved at a speed of 12 deg/s on a black background (0.16 cd/m^2^) toward either northeast or northwest direction. Given the dot speed and monitor refresh rate, the spatial displacement between consecutive dot frames, a critical parameter for motion perception [19], was 12 (deg/s)/20 (frame/s) = 0.6 (deg/frame) for each dot set. Dot size and density are 3×3 pixels (∼0.1° visual angle) and 16.7 dots deg^−2^ s^−1^, respectively. All dots were displayed within an invisible 20°-diameter circular aperture centered on the screen.

In the letter identification test, each English character was constructed from a matrix of 5×5 pixels (∼0.2° visual angle) and embedded on a 1° gray disk (18.52 cd/m^2^) at the screen center to mask motion dots in the background.

The experiment comprised seven one-hour sessions that were conducted for seven consecutive days. The second to sixth sessions employed the letter identification task for exposing background motions to participants. The first and last sessions used the motion sensitivity task to measure task-irrelevant perceptual learning as a result of passive exposure to weak motion signals. All sessions were conducted in a dimly lit room.

### Motion Sensitivity Task

**Figure 2:**
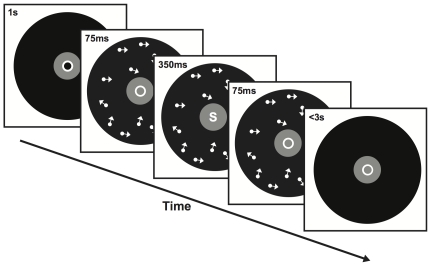
In a training trial, an observer identified central letter while being exposed to weak motion signals. Bright and dim letters were presented in alternating blocks and paired with different motion directions, respectively.

The motion sensitivity task required participants to detect whether a random dot display consisted of any coherent motion such as a meteor shower (Figure 2). The coherence level, namely the proportion of dots that moved coherently toward a randomly designated direction, was randomized from trial to trial. In each trial, a random dot motion (RDM) display was presented for 500 ms followed by a delay period of 500 ms. Then a Yes/No response for motion detection was recorded within 3 s. The next trial began after an interval of 500 ms during which a 0.1° fixation point appeared at the screen center.

Two motion directions (northeast and northwest) and six coherence levels (5%, 10%, 15%, 20%, 25%, 30%) were tested. There were 50 trials for each of the twelve conditions, forming 600 signal trials in total. To counterbalance signal trials, 600 independent noise (i.e., 0% coherence) trials were introduced. Then, a total number of 1200 trials were intermixed into 10 blocks with a self-paced break between blocks. A session took approximately 50 minutes to complete, and no feedback about task performance was given.

### Letter identification Task

**Figure 3:**
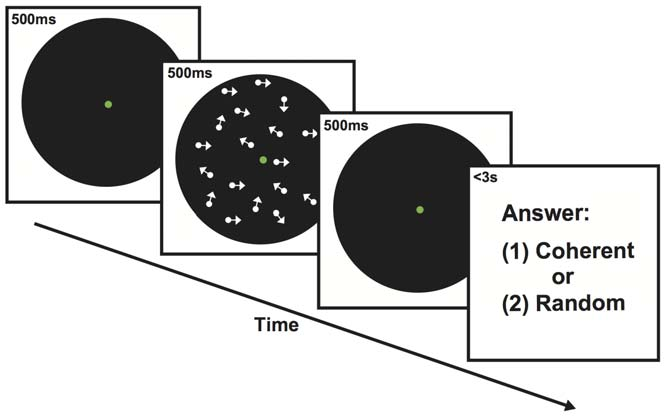
In a testing trial, an observer judged whether moving dots constituted any coherent motion. The motion coherence and direction were randomly varied from trial to trial.

The letter identification task required participants to key in an English letter that was flashed for 350 ms at the fixation in each trial (Figure 3). Each letter presentation was temporally centered in a 500-ms RDM display of 5% coherence, followed by a response period up to 3 s. The next trial is preceded by a 1-s interval during which a fixation circle of the letter size appeared in the center. Letters were randomly chosen from the English alphabet except (‘C’, ‘D’, ‘L’, ‘O’, ‘U’, ‘V’), whose hollow centers might invite diffuse rather than focal attention. Therefore, each trial was essentially a 20-alternative forced-choice task.

Two conditions of task difficulty were tested in a within-subject design. The luminance of letters was high (108.4 cd/m^2^) in the easy condition, and low in the hard condition (21.66 cd/m^2^). Each condition was paired with a particular RDM direction, which was randomly designated to each subject yet fixed throughout the study. In a daily session, 380 trials were grouped into 5 blocks for each condition, and two blocked conditions were interleaved with a self-paced break between blocks. A session took approximately 45 minutes to complete, and no feedback about task performance was given.

## Results

### Experiment 1

We used a dual-task RSVP design to examine how task difficulty affects memory of concurrently presented stimuli. Because observers' attention might shift back and forth between the central letters and background scenes and our main interest is their attentional deployment in face of a number target, the scene recognition performance reported here was averaged from 100 trials where the inside-RSVP scene probe was a target background.

**Figure 4:**
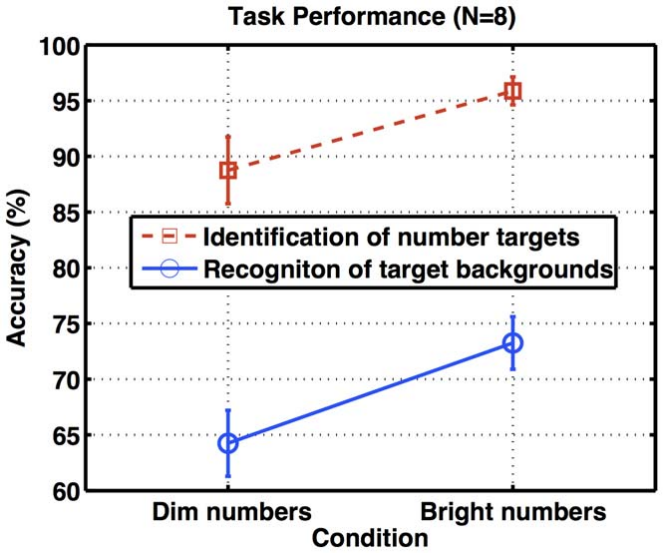
Task performance. The number identification and scene recognition accuracy (y-axis) are averaged from all participants, and plotted against two conditions of task difficulty (x-axis). Error bars indicate standard errors of group means.

The number identification and scene recognition results are summarized in Figure 4. Identification accuracy for dim numbers was significantly lower than that for bright numbers (paired-sample t-test: t(7) = −2.1537, p<.0341), indicating a successful manipulation of task difficulty. For recognition of target backgrounds, performance of the dim-target condition was also worse than that of the bright-target condition (paired-sample t-test: t(7) = −6.8739, p<.000118).

### Experiment 2

**Figure 5:**
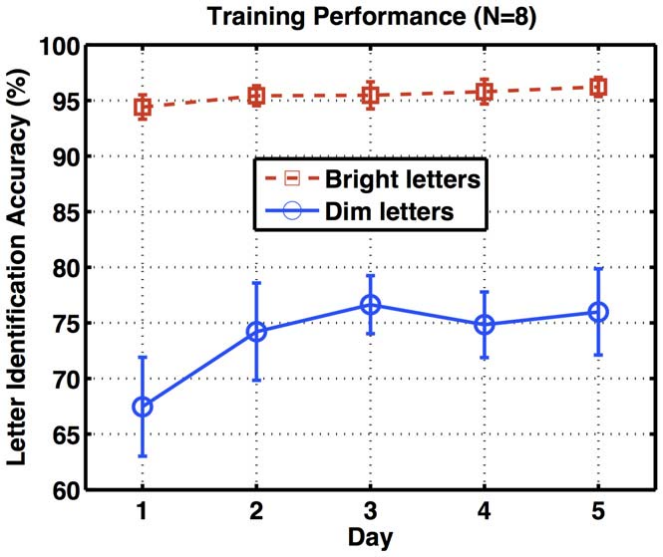
Training performance. The letter identification accuracy (y-axis) is averaged from all participants, and plotted against training day (x-axis). Error bars indicate standard errors of group means.

During the 5-day training phase, participants were asked to identify either bright or dim English letters at fixation while being exposed to weakly coherent random dot motion displays as task-irrelevant backgrounds. Figure 5 shows a steady progression of task-relevant learning on letter identification over five days of training. For bright letters, the identification performance on Day 5 was slightly better than Day 1 (paired-sample t-test: t(7) = 1.7295, p<.0637). Such an improvement from task-relevant learning was more pronounced for dim letters (paired-sample t-test: t(7) = 3.2927, p<.0066). More importantly, identification performance for dim letters was significantly lower than that for bright letters across days (paired-sample t-tests: t(7) = −5.4983, p<.000454 for Day 1; t(7) = −5.2392, p<.006 for Day 2; t(7) = −6.092, p<.000247 for Day 3; t(7) = −6.4105, p<.000182 for Day 4; t(7) = −5.1652, p<.000651 for Day 5), indicating a successful manipulation of task difficulty.

**Figure 6:**
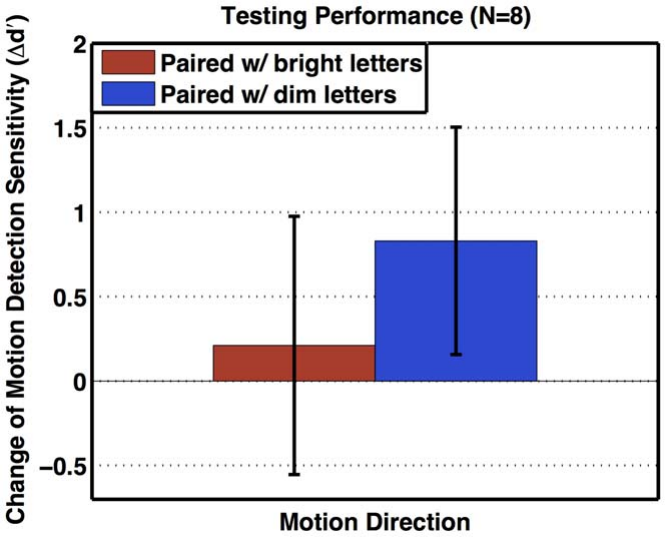
Testing performance change. The change of motion detection sensitivity (y-axis) is calculated by subtracting post- from pre-training sensitivity to each motion direction (x-axis), and averaged over all participants. Error bars indicate standard errors of group means.

To measure the effect of task difficulty on TIPL, we compared participants' pre- and post-training detection performances on two motion directions, each of which was paired with a different difficulty level during training. In the pre-training test, the eight participants showed comparable detection sensitivities for both motion directions (group mean ± standard error in d′: 1.81±0.15 for the bright-letter direction; 1.74±0.21% for the dim-letter direction) when performances of different motion coherence levels were averaged. The result from signal detection analysis is shown in Figure 6. The participants' decision criteria of detecting coherent motions (i.e., response bias “c" in signal detection theory) did not change significantly after 5 days of training on letter identification (paired-sample t-tests: t_pre-post_(7) = −1.1056, p<.3055 for the bright-letter direction; t_pre-post_(7) = −0.596, p<.57 for the dim-letter direction). However, against the limited-capacity attentional principle, hard/dim letters induced stronger TIPL than easy/bright letters (paired-sample t-test: t(7) = 1.9034, p<.0494).

## Discussion

Previous studies that used tasks similar to Experiment 1 had observed an attentional boost effect: an occasionally appeared target facilitated memory recognition of items that were temporally coincident with the target [20], [21]. The effect stands in sharp contrast to findings that more than one stimulus or task at a time interferes with each other owing to their competition for attentive processing and storage in the brain [11], [22]–[25].

However, in Experiment 1 we found that a harder central task led to worse memory recognition of a background scene. A harder central task appeared to demand more attentional resources. Consequently, recognition of less attended backgrounds was impaired. Such a result exhibits dual-task interference and is consistent with the spotlight/zoom-lens model in the attention literature.

Because attention is often reported to be limited in capacity, performing a perceptually difficult task is expected to demand substantially focused attention and thus narrow down the attentional spotlight. As a consequence, task-irrelevant features in the visual periphery may not be processed by the brain for being outside the coverage of the attentional spotlight.

Surprisingly, in Experiment 2 we found that a harder central task led to a larger magnitude of task-irrelevant learning of peripherally exposed signals. In other words, the popular spotlight/zoom-lens model in the attention literature cannot fully account for visual plasticity.

Visual attention to central stimuli impaired memory of background scenes in Experiment 1 but facilitated learning of background motions in Experiment 2. Thus, allocation of visual attention among stimuli is not always a zero-sum process as commonly believed. Note, however, that unlike other experiments whose task-irrelevant stimuli were clearly visible (i.e., supra-threshold), the task-irrelevant motion signals in Experiment 2 were perceptually weak (i.e., peri- or sub-threshold) and likely to escape from attentional regulation in the first place [26]. Consequently, these background motion signals might be learned, through target-triggered internal reinforcement signals [27], as target-associated contexts [16] rather than attention-competing distractors. In such a case, harder targets might induce stronger internal reinforcement signals because of greater arousal [28] or uncertainty [29] during task processing. Accordingly, reinforcement learning of task-irrelevant features could be strengthened by harder targets, as we observed in Experiment 2.

In daily life, there are lots of attention-competing objects surrounding us. When we watch TV or browse a web page, running advertisements on the sides battle for our attention with the target content at the center. When we drive on the street, moving scenes in the visual periphery grab our attention away from road signs or traffic lights up front. According to the result of Experiment 2, in some scenarios the more we pay attention to the central targets in view, the more we unconsciously pick up peripheral “distractors" as environmental contexts.

The positive correlation between task difficulty and task-irrelevant learning bears important implications for research in both attention and learning. First, when a visual task demands more attention, at least in some cases the brain may adaptively harness previously unengaged neural resources for more deliberate processing of all visual inputs, as opposed to just reallocating limited-capacity attention among task-relevant and task-irrelevant features. Second, task difficulty can be a critical factor that modulates or even enables subliminal task-irrelevant learning. To optimize exposure-based learning [30], [31], one needs to consider not only the salience of task-irrelevant features [17] but also the difficulty of task-relevant components.

Overall, although attention is known to gate visual plasticity [32]–[34], our study manipulates attention allocation through task difficulty and suggests that task-irrelevant perceptual learning is not simply induced by spared task attention. Factors other than attention may also regulate visual perceptual learning. Nonetheless, it awaits further investigation to clarify what theses factors are and how these factors interplay with attention in gating visual plasticity.
